# The Effect of Discordance Between Felt Age and Ideal Age on Subjective Well-being

**DOI:** 10.1093/geroni/igaf122.3079

**Published:** 2025-12-31

**Authors:** Isu Cho, Hyung In Park

**Affiliations:** Sungkyunkwan University, Seoul, Republic of Korea; Sungkyunkwan University, Seoul, Republic of Korea

## Abstract

The discrepancy between felt age and ideal age is termed as subjective age discordance (SAD). According to self-discrepancy theory, individuals tend to feel frustrated from experiencing discrepancies between how they perceive themselves and what they desire, which in turn can be associated with decreased well-being. Based on this theory, SAD is proposed to capture the status quo of individual well-being, separate from the discordance between felt age and chronological age that previous literature has been extensively tested. The current study examined the relationship between SAD and indicators of subjective well-being (i.e., life satisfaction, depression, and self-reported physical health) using polynomial regressions and response surface graphs. Data were collected from 318 Korean adults (aged 40 to 79) who responded twice with a 3-month interval. Results showed that a greater felt age against ideal age in Time 1 was linked to decreased life satisfaction in Time 2, whereas a greater ideal age against felt age was associated with enhanced life satisfaction, aligning with self-discrepancy theory. Plus, the greater SAD was in both directions, the higher depression was. However, SAD did not predict physical health. Besides, these patterns were moderated by chronological age such that the effects of SAD on life satisfaction and physical health were more prominent in younger adults (aged 59 or below, n = 164) than in older adults (aged 60 or above, n = 154). The findings suggest that older adults’ better socioemotional abilities (e.g., emotion regulation skills) may alleviate the negative effect of SAD on life satisfaction.

